# Possible role of negative human papillomavirus E6/E7 mRNA as a predictor of regression of cervical intraepithelial neoplasia 2 lesions in hr-HPV positive women

**DOI:** 10.1186/s12985-022-01822-1

**Published:** 2022-05-27

**Authors:** Maria Teresa Bruno, Nazario Cassaro, Salvatore Giovanni Vitale, Arianna Guaita, Sara Boemi

**Affiliations:** 1 Department of General Surgery and Medical Surgery Specialties, Gynecological Clinic, University of Catania, Catania, Italy; 2 Gynecological Oncology, Humanitas, Catania, Italy; 3 Multidisciplinary Research Center in Papillomavirus Pathology, University of Catania, Catania, Italy; 4 Department of Statistics, Sapienza University of Roma, Rome, Italy

**Keywords:** CIN2 regression, p16 HIC, E6/E7 mRNA, HSIL

## Abstract

**Background:**

The aim of this study was to evaluate the regression rate of CIN2 p16 positive lesions in women over 25 years of age and identify possible predictors of regression.

**Methods:**

A total of 128 CIN2 p16 positive patients over 25 years old were considered. The women met the following inclusion criteria: HPV genotype 16, 18, 31, 33, 45 positive, HPV E6 / E7 mRNA test positive, without immune system pathologies, not pregnant and had completed at least two years of follow-up. At each follow-up examination patients were examined by colposcopy, HPV test, E6/E7mRNA, targeted biopsy and p16 protein detection. The final state after the two years of follow-up was classified as progression if the histology showed a CIN3, persistence if the lesion was a CIN2, regression if negative or LSIL. The predicted regression factors evaluated were: HPV E6/E7mRNA, protein p16.

**Results:**

Overall, we had 35.1% (45 cases) of progression to CIN3, 41.4% (53 cases) of persistence and 23.4% (30 cases) of regression. The regression rate was higher in women with negative mRNA 92.8% (26/28), OR 312 (34.12–1798.76) *p* = 0.0001, while women with p16 negative had a regression of 22.6% (7/31), OR 0.94 (95% CI 0.36–2.46), *p* was not significant. We found no significant difference in regression between p16 positive (23.7%) and p16 negative (22.6%) CIN2 p16 lesions. p16 had a VPN of 22.6 (CI 95% 0.159–0.310), indicating that a p16 negative lesion does not exclude a CIN2 + .

**Conclusions:**

We had a regression rate of 23.4%, which was low if we consider that in the literature the regression rates vary from 55 to 63%. The discrepancy in the results may indeed be explained by the fact that all lesions in our study were hr-HPV positive and belonged to “older women” reflecting a more "high-risk" population. As regression factors we studied p16 and HPV E6/E7 mRNA. The results of our study show that HPV mRNA, if negative, appears to be able to identify CIN2 lesions with a higher probability of regression and underlines how a p16 negative is not an indicator of regression.

## Background

Richart's concept [1] of a step-by-step carcinogenesis from CIN1 to CIN2 to CIN3 belongs to an era in which HPV (Human Papilloma Virus) had not yet been correlated to cervical cancer and there was a static conception of preneoplastic cervical lesions (CIN) that inevitably had to progress to carcinoma. Today we know the oncogenic activity of papillomavirus [2, 3] and it is known that HPV-induced carcinogenesis is a rather slow process, characterized by a first phase of persistent HPV infection, followed by viral DNA integration and transformation of the squamous cells into CIN; the transformation of CIN3 into invasive cancer.

It is true that it is a process that takes many years, but it is also true that the process is reversible at any pre-invasive stage. The study by Oster [4] highlights the concept of CIN regression; CIN lesions are dynamic in the sense that all CINs can regress. In the literature about 60% of grade 1 cervical intraepithelial neoplasms (CIN1) regress, 30% persist, 10% progress to CIN3 and 1% progress to invasive cancer [5].

Therefore the concept of a preneoplastic lesion becomes dynamic and related to papillomavirus, thus in March 2012 the LAST Project (Anogenital Squamous Terminology) [6], consisting of members of the college of American pathologists and the American society of colposcopy, advised the use, for the related HPV pathology of the cervix, of the term SIL (Squamous Intraepithelial Lesion) instead of CIN, already used for cytology in the Bethesda System. It calls for abandoning of the three-level classification of CIN (CIN1, CIN2, CIN3) and opens up to a two-level terminology, which better reflects the biology of viral infection, LSIL\CIN1, and HPV viral infection, to be used at follow-ups and HSIL\CIN3, the true preneoplastic lesion, worthy of excisional treatment.

In the CIN classification there is also the difficulty in the interpretation of the various histological pictures, in fact, while pathologists agree when there are findings of carcinoma and it is easy to diagnose the histological CIN3, the CIN2 category continues to be an equivocal diagnosis, because it is not easily reproducible. Since the diagnosis of CIN2 cannot be reliably differentiated only by histopathological criteria, it is recommended to test for the immunohistochemical p16 protein (IHC). Negative CIN2 p16 is considered a viral lesion (CIN1), thus it undergoes follow-up, CIN2 p16 positive is considered a preneoplastic lesion (CIN3) and is treated with excision.

There is evidence that CIN2 has a regression rate of about 60% [7], especially in the category of young women (< 25 years old) [8, 9].

Current knowledge on CIN2 regression rates in women over the age of 25 is scarce [10].

The aim of this study was to evaluate the regression rate of CIN2 p16 positive lesions in women over 25 years of age and identify possible predictors of regression.

## Materials and methods

We studied the clinical files of 210 patients who, from April 2017 to April 2019, had undergone a biopsy for CIN2 at the Colposcopy clinic of the Gynecology Unit of the University Hospital of Catania (University of Catania, Italy) and we selected the women who met the following inclusion criteria: age over 25 years, histological diagnosis of CIN2, who preferred pending management rather than immediate excisional treatment, with squamous-columnar junction visible on colposcopy, positive p16 protein test, HPV DNA positive at genotypes 16, 18, 31, 33, 45, HPV E6 / E7 positive mRNA, not pregnant, without immune system disease and who had completed at least two years of follow-up.

The mean age of the HSIL/CIN2 patients included in the study was 38 years (range, 26–46). The study was conducted in accordance with the 1975 Helsinki Declaration. The investigations were conducted through the retrospective review of the medical database. The study protocol was notified, according to the current legislation on observational studies provided by AIFA, to the Catania1 Ethics Committee of the Catania university hospital, which did not request additions or changes to the protocol. Furthermore, the Catania1 Ethics Committee found the consent of the study participants unnecessary as the study concerned only the retrospective review of the medical database.

Follow-up was carried out every 6 months. At each follow-up examination patients were examined by colposcopy, HPV test, E6/E7mRNA, targeted biopsy and p16 protein detection. After cytological sampling for HPV DNA, samples were sent to the laboratory for DNA extraction and viral DNA genotyping by genetic amplification followed by hybridization with genotype-specific probes capable of identifying most of the HPV genotypes of the genital region [28 high-risk HPV genotypes (16, 18, 26, 31, 33, 35, 39, 45, 51, 52, 53, 56, 58, 59, 66, 68, 73, 82), low-risk (6, 11, 40, 43, 44, 54, 70) and undefined risk (69,71,74)]. The commercial method used was the MAG NucliSenseasy system (bioMerieux SA, Marct l'Etoile, France). The expression of viral oncogenes E6/E7 was investigated, identifying mRNA by the NucliSENS EasyQ HPV assay (bioMérieux). E6/E7 mRNA was tested for 16, 18, 31, 33, 45 genotypes.

Colposcopy was performed using a Zeiss OPM1F colposcope (Carl Zeiss, Jena, Germany) and applying acetic acid and Lugol iodine solution. Any colposcopic anomaly was classified according to the nomenclature proposed by the International Federation for Colposcopy and Cervical Pathologies (IFCPC) into 3 degrees of increasing abnormalities according to severity: Abnormal Transformation Zone (ATZ) grade 1 (ATZ1), grade 2 (ATZ2) or cancer. We evaluated the visibility of the squamous-columnar junction and specific biopsies were taken from the portio.

All histological specimens were fixed in 10% neutral buffered formalin and embedded in paraffin following routine procedures. The histological diagnosis of HSIL/CIN2 was established according to the WHO criteria on the basis of morphological criteria using hematoxylin and eosin (H&E) staining, without knowing the hrHPV status or the Pap test result. All slides were reviewed by two gynecological pathologist.

And then a test for the p16 protein with immunohistochemical techniques (IHC), using p16 markers (CINtec p16 INk4a Histology Kit, clone E6H4) was carried out.

The final state after the two years of follow-up was classified as progression if the histology showed a CIN3, persistence if the lesion was a CIN2, regression if negative or LSIL. Cases of progression underwent LEEP.

The predicted regression factors evaluated were: HPV E6-E7mRNA, p16protein.

### HPV testing: NucliSENS EASYQ HPV assay (bioMérieux)

The technique was previously described [11].

### HPV E6/E7 mRNA testing: PreTect HPV-proofer real-time multiplex NASBA test

A detailed description of the HPV-Proofer protocols has been published [12].

The amplification and detection of HPV E6/E7 mRNA was performed with the PreTect HPV-Proofer real-time multiplex NASBA test**,** using PCR with primers/probes for HPV types 16, 18, 31, 33 and 45.

The PreTect HPV-Proofer real-time multiplex NASBA test was performed as suggested by the manufacturer (NorChip AS, Klokkarstua, Norway). Briefly, three premixes were made by reconstituting the reagent sphere, containing nucleotides, dithiothreitol and MgCl2, in a reagent sphere diluent (Tris–HCl, 45% dimethyl sulfoxide). Then, the primer-molecular beacon mixture U1 small nuclear protein specific ribonucleoprotein A (U1A) -HPV-16, HPV-33-HPV-45 or HPV-18-HPV-31 was added along with a KCl stock solution. Ten microliters of this premix were distributed to each well in a reaction plate, followed by the addition of RNA and 4 min of incubation at 65 °C (to destabilize secondary RNA structures) and 4 min of incubation at 41 °C. The reaction was initiated by adding enzymes (avian myeloblastosis virus reverse transcriptase, RNase H and RNA polymerase T7) and was measured in real time using a Lambda FL 600 fluorescence reader (Bio-Tek, Winooski, VT) at 41 °C for 150 min. The total reaction volume was 20 μl. A newly developed software package (PreTect analysis software; NorChip AS, NO) was used for the analysis of the experimental data. The excitation filters (nm) for 6-carboxyfluorescein and Texas Red were, respectively, 485/20 and 590/20 and the emission filters λ (nm) were, 530/25 and 645/40, respectively. RNA isolated from CaSki cells was used as a positive control for HPV-16. Artificial and standardized oligonucleotides corresponding to the viral sequence were used as positive controls for HPV types 18, 31, 33 and 45. As a performance control, to avoid false negative results due to RNA degradation, we used a set of primers and a probe directed against human U1A mRNA. Negative controls consisting of all reagents except RNA were included in each run.

### Immunostaining slides for p16: CINtec® INK4a; Roche diagnostics

Tissue sections obtained from biopsy or cervix cone following LEEP were paraffin embedded and formalin-fixed and were then dewaxed in xylene hydrated using graduated ethanol blends. Slides were treated with 0.3% hydrogen peroxide for 30 min to quench endogenous peroxidase activity, rinsed for 20 min with PBS buffered saline. A monoclonal mouse antibody directed against human p16INK4a protein (clone E6H4) was used and after an overnight incubation at 4 °C, a goat anti-mouse secondary antibody, was applied for 30 min at room temperature, followed by the avidin–biotin-peroxidase complex (Vector Laboratories, Burlingame, CA, USA) for another 30 min at the same temperature. The slides were then incubated with DAB (3, 3'-diaminobenzidine) for 5 min, counterstained with hematoxylin for 5 s, and then viewed under a microscope (Carl Zeiss, Germany).

For the results of p16 we considered two factors: intensity and distribution. Intensity is considered as diffuse block, patchy or focal; the distribution may be limited to a lower third, up to the middle third or up to the upper third.

p16 was positive if the samples showed a continuous nuclear or nuclear and cytoplasmic staining of the cells of the basal and parabasal layer of the epithelium, extended to more than a third of the entire epithelial thickness, and exclusively intense localization.

p16 was negative if the samples showed the absence of staining in the epithelium, cytoplasmic staining of isolated cells or small focal cell clusters, and an extent of less than one third of the epithelial thickness.

p16 is not recommended in cases of unambiguous histological lesions. Therfore, if a CIN1 lesion shows diffuse and strong p16 according to LAST, these lesions have yet to be interpreted as CIN1, despite p16 being diffuse.

Some cases that were negative for the p16 protein during follow-up were classified as CIN2 p16 negative because they still had a convincing CIN2 morphology and did not fall within the current definitions of LSIL.

We did not consider the viral genotype as all the women in the study were hr-HPV positive at baseline for genotypes 16, 18, 31, 33, 45.

### Statistical analysis

Statistical analysis was performed by using the SPSS software package for Windows (version 15.0, SPSS, Chicago, IL, USA). Descriptive statistics are expressed as frequency, arithmetic mean, and percentages. The results are summarized in tables. The relationship between the categorical variables was evaluated by Chi-square tests or exact Fisher tests, depending on the sample size. We studied p16 and HPV E6/E7 mRNA as predictive variables, their ability as predictors of regression or progression were evaluated by estimating odds ratios (OR) with confidence intervals (CI) of 95%. *p* < 0.05 was considered statistically significant.

In addition, the sensitivity, specificity, negative predictive value (NPV) and positive predictive value (PPV) of the HPV E6/E7 mRNA test and p16 were evaluated.

## Results

Of the 210 patients with a histological diagnosis of HSIL/CIN2, 128 met the inclusion criteria. After a follow-up period of 24 months, we had 35.1% (45 cases) progression to CIN3, 41.4% (53 cases) persistence and 23.4 (30 cases) regression (Table 1).

**Table 1:** Regression, Persistence and Progression according to variables: p16 and E6/E7 mRNA

Variables	n°	Regression	Persistence	Progression	*p*-value
		n (%)	n (%)	n (%)	
	128	30 (23.4)	53 (41.4)	45 (35.1)	
*p16*					
negative	31	7 (22.6)	21 (67.7)	3 (9.7)	0.078
positive	97	23 (23.7)	32 (33)	42 (43.3)	
*E6/E7mRNA*					
negative	28	26 (92.8)	2 (7.14)	0	0.0001
positive	100	4 (4.0)	51 (51)	45 (45)	

No case of progression to invasive carcinoma was observed.

**Fig. 1:**
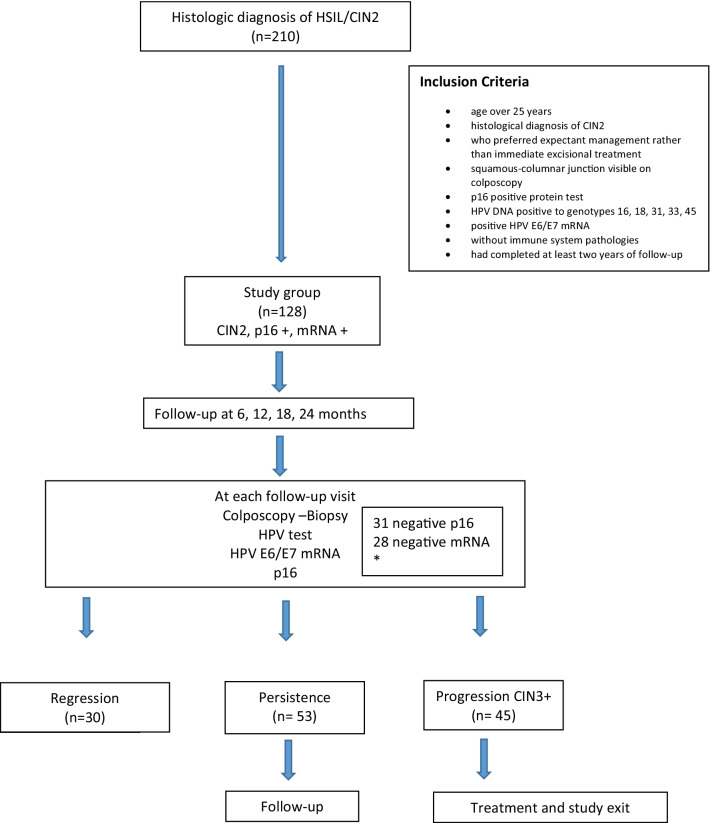
Flow diagram and final outcome of CIN2 patients. *During the 24-month follow-up, 31 positive p16 cases and 28 baseline-positive mRNA cases became negative

The flowchart for participation in the study is shown in Fig. 1.

The study group consisted of 128 women, p16 positive and mRNA positive at baseline.

During the 24-month follow-up, 31 positive p16 cases and 28 positive mRNA cases became negative.

Stratification of the study sample according to the results of mRNA and p16, after two years of follow-up, is shown in Table 1. In the negative p16 group we had 3 cases of progression to CIN3, OR 0.14 (95% CI 0.04–0.49) *p* < 0.05, 21 cases of persistence and 7 cases of regression, OR 0.94 (95% CI 0.36–2.46), p not significant. The positive p16 group had 23 cases of regression, 32 cases of persistence and 42 cases of progression, OR 7.13 (95% CI 2.03–25.91,) *p* < 0.05.

Of the mRNA negative women there were 26 cases of regression, OR 312 (95% CI 54.12%-1798.76), *p* < 0.05, no case of CIN3 progression and only 2 cases of persistence; the positive mRNA women had 4 cases of regression, 51 cases of persistence and 45 cases of progression, OR 22.91 (95%C I3.00–17.00) *p* < 0.05.

Table 2 shows that the regression rate was higher in women with negative mRNA 92.8% (26/28), OR 312 (95% CI 54.12–1798.76) *p* < 0.05, while women with negative p16 had a regression of 22.6% (7/31) OR 0.94 (95% CI 0.36–2.46), p was not significant. We found no significant difference in regression between p16 positive and p16 negative CIN2 lesions (*p* = 0.078).

**Table 2:** Odds Ratio and p-value of patients with CIN2 regression according to p16 and E6/E7mRNA at 24 months of follow-up

CIN2 Regression				
Variables	n°	n (%)	Odds Ratio (95% CI)	*p-value*
Overall	128	30 (23.4)		
*p16*				
negative	31	7 ( 22.6)	0.94 (0.36–2.46)	0.8971
positive	97	23 (23.7)	1.07 (0.41–2.79)	0.8971
*E6/E7mRNA*				
negative	28	26 (92.8)	312 (54.12–1798.76)	0.0001
positive	100	4 (4.0)	0.00 (0.00–0.02)	0.0001

The rate of progression in the two study populations differed little, 45% (45/100) of mRNA positive women, with OR 22.91 (95% CI 3.00–17.00), *p* < 0.05, compared to 43.3%(42/97) of p16 positive women, with OR 7.13 (95% CI 2.03–25.91), *p* < 0.05 (Table 3).

**Table 3:** Progression rate, odds ratio and p-value of CIN2 patients at 24 months of follow-up according to p16 and E6/E7mRNA

CIN2 Progression				
Test	n°	n (%)	Odds Ratio (95% CI)	*p-value*
	128	45 (35.1)		
*p16*				
negative	31	3 (9.7)	0.14 (0.04–0.49)	0.0006
positive	97	42 (43.3)	7.13 (2.03–25.04)	0.0006
*E6/E7mRNA*				
negative	28	0	0.04 (0.01–0.33)	0.0001
positive	100	45 (45)	22.91 (3.00–175.00)	0.0001

Table 4 shows the values of sensitivity, specificity, positive predictive value (PPV) and negative predictive value (NVP) for p16 and for mRNA E6/E7.

**Table 4:** Performance indicators of p16 and E6/E7 mRNA

	Sensibility (%)	95% CI	Specificity	95% CI	PPV	95% CI	NPV	95% CI
p16	75	0.670–0.825	23.3	0.165–0.318	76	0.678–0.825	23	0.159–0.310
E6/E7 mRNA	98	0.932–0.995	86	0.793–0.918	96	0.905–0.985	93	0.866–0.955

## Discussion

Conducting long-term prospective observational studies is impossible for patients with CIN3, for obvious ethical reasons. The New Zealand study [13] was the only prospective study on the natural history of cervical cancer in which women with CIN3 were discontinued from treatment. Over 30% of these women developed cervical cancer, compared to less than 1% of treated women. This study, a milestone in the natural history of cervical cancer, showed us that it takes at least 10 years for progression from CIN3 to cancer. Unfortunately, no data on CIN regression were obtained, thus the data we have today on CIN3 regression are derived from retrospective studies, since for ethical reasons CIN3 must necessarily be treated. Oster's study [4] provides evidence of the regression capacity of CIN3 of 33% and CIN2 of 55%.

A recent systematic review and meta-analysis found a CIN2 regression rate after 6 months of 52% and a regression rate of 50% after 24 months. Regression is more frequent for women under the age of 30 [14].

In a prospective cohort study [15] of 95 women aged 18 to 23 years, the regression rate was 63% within 2 years.

Considering that the elimination rate of HPV infection is higher in younger women and the persistence of this infection seems to increase with age, CIN 2 lesions are less likely to regress with increasing age [16, 17].

Few studies have evaluated the CIN2 regression rate in women over the age of 25.

In our study we only considered women over the age of 25 and the regression rate was 23.4% (30/128).

We had a low regression rate, considering that previous studies had regression rates from 55 to 63%.

The discrepancy in the results can be explained by the fact that all the lesions in our study were hr-HPV positive and belonged to “older women” who reflected a more "high-risk" population. The study by Discacciati also included hr-HPV negative lesions or lesions with unknown HPV status and it found no association between age and CIN2 regression [18]. Miyamoto's study, which included older women, whose mean age was 36.5–39.8 years, found a higher mean age in women who showed disease regression than progression and persistence [19]. Based on these results, it is not possible to establish an association between age and CIN2 regression. A biological explanation for this could be that the elimination of the disease is an immune-mediated process, which can be independent of age and duration of the HPV infection.

It has been established that CIN2 has spontaneous regression capabilities, therefore it becomes important, despite LAST recommending a two-level system, to maintain the CIN2 category for better clinical management; for example, allowing conservative treatment of CIN2 lesions in young women, and it would be useful to identify CIN2 lesions capable of regressing.

To date, no test and no biomarker can predict the progression or regression of CIN2. HPV DNA tests and PCR genotyping are very sensitive but not very specific, the presence of HPV DNA does not discriminate active infections from latent or transient ones [20]. PCR genotyping highlights the presence of high-risk genotypes, in particular genotype 16, the most endowed with oncogenic capacity [21, 22]. One of the ways to increase specificity for the detection of high-grade cervical disease and identify markers of its evolution, is to look for the expression of viral oncogenes E6/E7 [23], by directly testing E6/E7 mRNA in the lesion (HPV E6/E7 mRNA test) and indirectly by dosing the p16 protein, Kinase-Cyclic dependent inhibitor (p16 protein test): its increase indicates that the virus's E7 oncogene degraded the pRb protein by precipitating the cell towards oncogenesis. The two most promising predictors are: p16 IHC and HPV E6/E7 mRNA.

The efficacy of p16 as a diagnostic biomarker in HSIL lesions (CIN2) has been widely demonstrated [24]. The use of p16 helps to distinguish CIN3 from its imitations, such as immature squamous metaplasia or therapeutic changes [25], but its sensitivity in detecting CIN2/CIN3 may be reduced by a small fraction of CIN 2/3 or carcinomas that may show weak or negative p16 staining [26].

Diffuse p16 IHC staining is typical of CIN3, but cases of CIN2 in which the positivity to p16 is not total, also lend themselves to creating false negatives or positives.

LAST's latest recommendation states that p16 should not be used in cases with unique morphology. Up to 35% of CIN2 histological lesions are p16 negative i.e. not broadly positive but morphologically retain the characteristics of a CIN2, in addition, up to half of CIN1 lesions are largely p16 positive [27–29] (destined to progress). In our study of 31 CIN2 cases that became negative for the p16 protein during follow-up, they were classified as CIN2 p16 negative because they still had a convincing CIN2 morphology and did not fall under the current definitions of LSIL (of these only 7 also regressed morphologically).

The efficacy of p16 as a prognostic biomarker in CIN2 is currently not evaluated due to a limited number of studies and their conflicting results [30].

Logic tells us that a negative p16 or a negative mRNA should predict a favorable prognosis for CIN2 lesions towards regression. But it is not that simple, it has been shown that a positive p16 CIN is less likely to regress than a negative p16 CIN and is more likely to progress. The difficulty in applying these results to clinical practice is that some positive p16 CINs may still regress, while some negative p16 CINs may still progress.

Analyzing the data related to the negative state of the two markers during the follow-up we had conflicting results. mRNA test negativity is consistent as a marker of CIN2 regression with 92.8% of regression cases, OR 312 (95% CI 54.12%-1798.76), *p* < 0,05*,* while p16 negative with 22.6% of regressed cases, OR 0.94 (95% CI 0.36–2.46) *p* = 0.897, seems unable to predict lesion regression.

Branca [24] noted that HPV HR clearance was slightly faster in p16INK4a positive women than in negative women, but the difference was not significant. These data imply that p16INK4a does not predict clearance (p 0.198) or persistence (p 0.243) of HR-HPV in the cervix.

Guedes saw that p16 negative women had a slower spontaneous CIN2 regression, compared to p16 positive women, indicating that p16 does not predict CIN2 outcome.

In our study, negative p16 women regressed by 22.6%, OR 0.94 (95% CI 0.36- 2.46), p was not significant, and this was in agreement with Guedes' study [29] we found no significant differences in regression between p16 positive and p16 negative lesions (Table 3).

Our results confirm those of other authors [24, 27, 31] and underline how a p16 negative is not an indicator of regression.

In the p16 negative group, three women had progression to CIN3 during follow-up. This fact is confirmed in the literature. Genoves [32] reported that a small percentage of negative CIN2 p16 lesions may progress to CIN3, in particular, 5 out of 6 cases of negative CIN2 p16 biopsies demonstrated HSIL at follow-up excision, suggesting that a large percentage of negative CIN2 p16 may still progress to HSIL. Maniar found that 26.2% (27/103) of positive CIN2 p16 had progressed to CIN3, while only 4.4% (2/45) of negative CIN2 p16 had progressed to CIN3, a statistically significant difference [33]. Nishio examined the progression of CIN 1–2 and found 6 out of 66 cases of CIN 1–2 p16 negative were progressive [34].

A p16 negative does not exclude an HSIL/CIN3 outcome, in fact, in the study group we observed a low, but significant, number (3 cases) (*p* < 0.05) of patients with negative p16 staining who developed a CIN3 in the follow-up. On the other hand, p16 has a VPN of 22.6 (CI 95% 0.159- 0.310), indicating that a p16 negative lesion does not exclude a CIN2 + . These findings raise concerns about the usefulness of p16 as a marker of regression in women with CIN2 in clinical practice and reinforce LAST recommendations indicating that p16 should only be used in cases with equivocal CIN1/CIN2 characteristics or in the differential diagnosis between CIN2/3 and its benign imitators [35].

The rate of progression in the two study populations differed little, 45% (45/100) of mRNA positive women, OR 22.91(95% CI 3.00–17.00) *p* < 0.05, compared to 43.3% (42/97) of p16 positive women, OR 7.13 (95% CI 2.03–25.91) *p* < 0.05.

P16 has a good sensitivity (75.5%) and a good PPV (76.6%) and with an OR 7.13 it is a valid marker of progression: this has been confirmed in the literature.

The mRNA test we used in our study detects only 5 of the 18 types of high-risk HPV included in the DNA test. These 5 high-risk HPV types (16, 18, 31, 33 and 45) comprise 97% of the oncogenic HPV types found in cervical cancers in Europe and North America [22]. Typically, the sensitivity of NASBA E6/E7 tests is limited by the small number of targeted types of hr-HPV [36], in our study we considered only women positive for the genotypes covered by the NASBA test (5 genotypes) achieving good sensitivity and specificity of the test.

The HPV E6/E7 mRNA test has an excellent sensitivity (98%), an excellent specificity (86.7%), a PPV of 96% and an NPV of 93%. These values make mRNA an excellent marker of regression but also of progression in its positive form, OR 22.91(95% CI 3.00–17.00), *p* < 0.05.

Negative mRNA cases revealed 93% lesion regression, OR 312 (34.12–1798.76) p < 0,05, persistence 7%, and no cases of progression.

The results of the NTCC2 study [37] and the study by Lie et al. [38] show that HPV mRNA, if negative, seem able to identify CIN2 lesions with a higher probability of regression. Moreover, the study of PG Rossi et al. associates a greater capacity for regression with mRNA, in fact, the regression of CIN2 has been estimated to be 70% and 40% in E6/E7 mRNA and p16 negative women, respectively [39]. A negative HPV E6/E7 mRNA test confers a risk of invasive cervical cancer and CIN2 + at 5 years, comparable to that of a negative HPV DNA test [37].

These studies support our results and suggest that the mRNA of HPVE6/E7 may ultimately be higher than p16 in identifying cases destined to regress [40].

Authors [41] indicate that high sensitivity and negative predictive value, unlike p16 [29], can be used to predict recurrence of cervical lesions after LEEP. In our previous work [11] we showed how the persistence of genotype 16 after LEEP indicates a high risk of recurrence of high-grade lesions. In a persistent HPV16 woman, detection of a positive mRNA emphasizes the risk of recurrence.

Thus, there is evidence that the positive p16 protein is a valid marker of lesion progression [24] the same result was not shown as a regression marker [30] when p16 is negative. E6/E7 mRNA is an excellent marker of regression but also of progression in its positive form with an OR 22.91(95% CI 3.00–17.00), *p* < 0.05. The mRNA of HPV E6/E7 may ultimately be higher than p16 in identifying cases destined to regress [40]. This different ability of p16 and mRNA to be indicators of regression can be explained by mechanisms that induce silencing of the p16INK4a gene by methylation of its promoter and overexpression of the BMI polycomb-1 gene as already evidenced in carcinomas with p16 negative [42].

Oka et al. are the only authors who have investigated an association between viral DNA methylation and histologically confirmed CIN natural history. In his study (15 cases) there were 8 cases of CIN1/2 with progression to CIN3 and one case of CIN3 that regressed. Methylation rates of the L1 gene were significantly higher in the progression group [43], and it is in this direction that research is shifting, markers of epigenetic effects are among the most studied at the moment.

Our study has several strengths, not least the inclusion of a sample with restrictive inclusion criteria and a long 24-month follow-up performed by the operators themselves. In addition, all biopsies sampled inside or outside our department were reviewed by two pathologists to include only patients with "true" CIN2. The limitations of our research are mainly related to the small sample size and its retrospective nature. The retrospective nature did not permit the collection of all data such as the size of the initial lesion, the use of oral contraceptive pills, sexual practices and the use of drugs that can contribute to regression or persistence. Other factors that can influence the results may be the state of the host's immune system, which can have a significant influence on the elimination of the lesion and the reversal of the first oncogenic steps. This evidence could be increased by the fact that women underwent targeted biopsy every 6 months during follow-up. We cannot rule out that the biopsy may have stimulated the immune system and influenced the natural history of CIN2 causing its regression. Another limitation of the study is the lack of exposure of HPV typing data in relation to p16 and mRNA results for incomplete data [44]. Another possible limitation is the possible sampling errors of the histological sample and the same immunohistochemical technique that sometimes presents interpretative challenges. Differences in the interpretation of staining and in the orientation of sections are an inevitable limitation of these investigations. Further prospective studies with more patients are needed to confirm the current findings.

## Conclusions

We had a spontaneous regression rate of 23.4% of CIN2 in our study. The study sample, consisting of elderly hr-HPV positive women, is a high-risk population and explains the low regression rate, in contrast to the literature. We found no significant differences in regression between p16 positive and p16 negative CIN2 lesions.

There is evidence that the p16 positive protein is a valid marker of lesion progression, however, the same result has not been shown as a marker of regression when p16 is negative; unlike HPV E6/E7mRNA, which is very useful as a marker of progression when it is positive and regression when it is negative.

Our experience shows that negative mRNA E6/E7 is a valid prognostic marker of CIN2 regression and therefore reduces overdiagnosis and the consequent overtreatment [45].

## Data Availability

The datasets used and/or analyzed during the current study are available from the corresponding author on request.

## Abbreviations

HPV: Human papillomavirus
hr-HPV: High risk HPV
CIN2: Cervical intraepithelial Neoplasia grade 2
NASBA: Nucleic acid sequence-based amplification
IHC: Himmunohistochimical
